# The Effect of Ramadan Fasting on the Coping Strategies Used by Male Footballers Affiliated with the Tunisian First Professional League

**DOI:** 10.3390/healthcare11071053

**Published:** 2023-04-06

**Authors:** Jamel Hajji, Aiche Sabah, Musheer A. Aljaberi, Chung-Ying Lin, Lin-Yi Huang

**Affiliations:** 1 Higher Institute of Sport and Physical Education of Gafsa, Gafsa University, Gafsa 2100, Tunisia; 2 Faculty of Human and Social Sciences, Tunis University, Gafsa 2100, Tunisia; 3 Faculty of Human and Social Sciences, Hassiba Benbouali University of Chlef, Chlef 02076, Algeria; 4 Faculty of Medicine and Health Sciences, Taiz University, Taiz 6803, Yemen; 5 Institute of Allied Health Sciences, College of Medicine, National Cheng Kung University, Tainan 701, Taiwan; 6 Department of Pediatrics, E-Da Cancer Hospital, I-Shou University, Kaohsiung 824, Taiwan

**Keywords:** fast day, adaptation, lifestyle, athletes, men, mental health

## Abstract

This study aimed to discover coping strategies among professional male Tunisian footballers during the Ramadan 2021 fast. One hundred and eighty footballers who belong to twelve Tunisian professional clubs (age: 25.54 ± 4.41 years, weight: 77.19 ± 5.99 kg; height: 180.54 ± 7.28 cm; BMI: 23.67 ± 0.58) were tested during three sessions: one week before Ramadan, during the last week of Ramadan, and one week after Ramadan 2021. The footballers completed the Arabic version of the Inventory of Coping Strategies for Competitive Sport (ICSCS) scale in each session. Responses were recorded retrospectively one hour after a competition. The analysis of variance revealed a significant effect of Ramadan fasting on the adaptation profile of footballers (F = 3.51; *p*-value = 0.0001). Before and after Ramadan fasting, active coping dominates the adaptation profile of Tunisian professional footballers. During Ramadan, footballers use an irregular and unbalanced coping profile. The lifestyle change induced by the Ramadan fast significantly and negatively affected the adaptation profile of Tunisian professional footballers. Under the effect of the month of Ramadan, footballers developed a different coping profile from that of normal months.

## 1. Introduction

Ramadan fasting is one of the religious rituals of Muslims. It is a form of intermittent fasting, which is practiced by more than 1.5 billion Muslims annually throughout the month of Ramadan. Ramadan is the ninth month of the Islamic calendar. In this month, worldwide Muslims must fast for 29–30 days. The daily fasting duration in Ramadan may vary between seasons, from 12 to 18 h, depending on the season and geographical area [1,2]. During fasting, individuals do not eat anything from sunrise to sunset. From sunset to brightness, Muslims can eat freely. Hence, the time of sleeping and eating may be affected by Ramadan, as the frequency and quantity of food, the duration of night sleep, and sports activities are reduced [3,4].

Muslim athletes often encounter significant metabolic, behavioral, and dietary disruption when they engage in Ramadan fasting [5,6,7,8]. Athletes who fast during Ramadan can potentially confer hypohydration [9], disturbances in body mass [10,11,12,13], variations in aerobic qualities [14,15], intense anaerobic qualities [16,17], metabolic and hormonal disturbances [18,19], disorders in physical activity profile [20], sleep disorders including sleep deficit [21,22], mood swings, and general impairment of physical and psychomotor performance [5,23]. Accordingly, many studies have shown a negative effect of fasting in Ramadan on several aspects of physical performance [24,25]. For example, athletes experience varying stress levels during Ramadan due to the disruption and alteration of their biological clock [26,27,28].

Aside from the physical effects, mood disturbances with mental fatigue have also increased during Ramadan [16,24,25]. Several studies have shown the harmful effects of fasting in Ramadan on the mental aspects of performance [29,30,31,32,33]. For example, one study identified the adverse effects of Ramadan fasting on sleep and the performance of footballers participating in the 2012 Olympics [30]. Another study showed a negative impact of Ramadan fasting on cognitive performance [34]. The results of these studies suggest that decision-making behaviors during training and competition conditions may be negatively affected during Ramadan. Therefore, understanding how to minimize the adverse effects of Ramadan fasting on Muslim athletes is a crucial issue.

Some studies have designed mental preparation sessions for Muslim athletes before the beginning of the Ramadan fast to assimilate proactive coping strategies [24,25,29]. Experienced Muslim athletes, especially those with many years of sports experience, usually have developed strategies to cope with the Ramadan fast [30,35]. For example, they acquired learned behavioral patterns that they found effective in meeting the fasting challenge. Some intermittent practice fasting throughout the year as strategies to prepare for the month of Ramadan, which is characterized by continuous fasting, changes in eating behavior, physical activity, sleep, and emotional and cognitive behavior, and lower energy intake resulting from the restriction of feeding to a limited time [2,15,36]. The athlete’s nutritional status, degree of training, temperature, humidity, and daily fluid intake may reflect differences such as intermittent or continuous fasting [36,37,38]. Adaptation essentially involves the individual response of an athlete to cope with a stressful situation [39]. On the other hand, preventive coping aims to develop resources to reduce the effects of uncertain and stressful future events [25,40,41]. Proactive adaptation involves strategies for developing public resources to achieve personal goals.

Ideally, a male Muslim athlete immerses himself in competition to achieve the best performance. Although fasting during Ramadan represents an obstacle, he does everything to develop coping strategies to achieve his goals [24,25,29,30]. Some Muslim athletes adopt good coping strategies and have been successful in combating the negative influence of Ramadan fasting on their subsequent physical performance. In contrast, those who are less capable of coping with the disturbances induced by Ramadan fasting may not have peak performance [5,6,23].

In general, commonly used coping strategies include task-focused coping, in which the athlete directly confronts the threatening situation, and avoidance-focused coping, in which the athlete attempts to disengage or distract themselves from the stressful situation [42,43,44]. In addition, some studies consider fasting during Ramadan as an unfavorable condition for achieving the desired performance [5,6,7].

Therefore, it is important to understand the adaptation profile adopted by Tunisian professional footballers during the Ramadan fast. The present study aimed to examine the self-generated coping strategies adopted by our participants before, during, and after Ramadan. Specifically, Tunisian professional footballers, such as most Muslim athletes, fast every year during Ramadan. Nevertheless, they continue sports and physical activities and simultaneously cope with this stressful situation using self-developed coping strategies without prior mental preparation.

This study aims to examine the effect of Ramadan fasting on the coping profile of Tunisian professional footballers before, during, and after Ramadan. This study also aims to determine the coping strategies used by participants during the month of Ramadan.

Hence, the study hypothesized that; there is a significant effect of Ramadan fasting on the footballers’ coping strategies. It is expected that the results of this study will be a reference that will help coaches and mental trainers to improve the performance of their athletes.

## 2. Materials and Methods

### 2.1. Study Design

**Figure 1:**
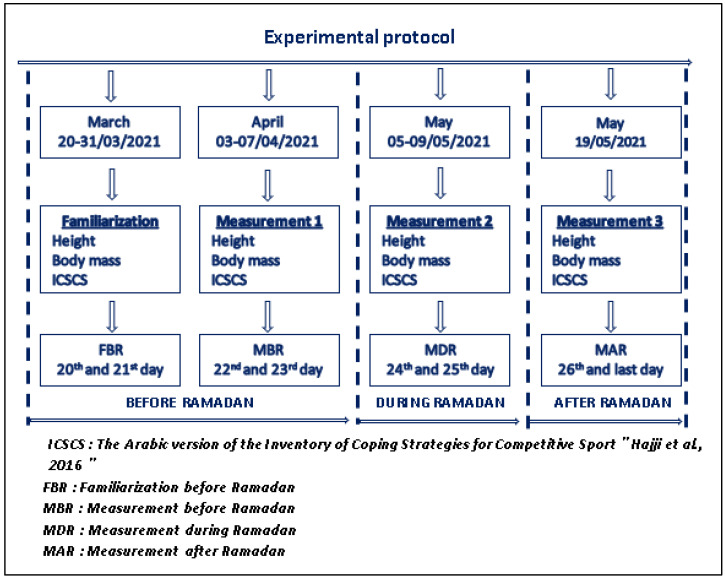
Experimental protocol adopted in the present study [45].

The study was conducted in Tunisia in 2021. Ramadan began on Tuesday, 13 April 2021, and ended on Wednesday, 12 May 2021. The duration of each daytime fast was approximately 16 h starting at ~3:22 a.m. and ending at ~7:20 p.m. After the approval of officials and technical staff, the footballers were first invited six weeks before Ramadan to familiarize themselves with all the procedures involved in the study (FBR: Familiarization Before Ramadan). Then, the fasted footballers from the respective clubs were asked to respond to the Arabic version of the Inventory of Coping Strategies for Competitive Sport (ICSCS) [45], and measure their height and weight for body mass index (BMI) calculation in three sessions. The first session was carried out a week before Ramadan (MBR) from 3–7 April 2021, following the 22nd and 23rd day of the Tunisian professional championship. The second session occurred in the last week of Ramadan from 5–9 May 2021 (MDR), following the 24th and 25th day. The previous session was carried out a week after Ramadan (MAR) on 19 May 2021, following the last day of the championship (Figure 1).

The study protocol was reviewed in-depth and fully approved by the “Ethics Committee for the Protection of Populations in the South” (C.P.P.SUD), Sfax, Tunisia (protocol reference C.P.P.SUD N 0031/2020). This study was based on the latest version of the Helsinki declaration and its subsequent amendments. The registration code for the trial is PACTR20211254567573.

### 2.2. Participants

After obtaining approval from club officials, coaches, and players, one hundred and eighty male footballers with a mean age of 25.54 years (SD = 4.41) volunteered to participate in this study. Fasting footballers were invited from 12 Tunisian clubs affiliated with Pro League 1 (CAB: Club Athlétique Bizertin; UST: Union Sportif de Tataouine; CA: Club African; ESS: Etoile Sportive du Sahel; ASS: Avenir Sportif de Soliman; CSS: Club Sportif Sfaxien; ESM: Etoile Sportive de Métlaoui; USB: Union Sportive de Ben Guerdane; USMO: Union Sportive de Monastir; ASR: Avenir Sportif de Rejiche; EST: Espérance Sportive de Tunis; OB: Olympique de Béja). These 12 clubs, among 14 clubs, regularly participate in the Tunisian first professional league. The same activity continued during Ramadan; these players were recruited to observe Ramadan fasting. The characteristics of the participants are shown in Table 1.

### 2.3. Applied Protocol and Measures

The Inventory of Competitive Sports Coping Strategies (ICSCS) (Supplementary Table S1) [43] was used to study the coping profile of Tunisian professional soccer players. The Gaudreau and Blondin [43] ICSCS is the most commonly used instrument in the sports context to assess coping strategies for competitive sports [44]. Due to the cultural specificity of the present sample, we used the Arabic version of ICSCS [45], which has been validated according to the methodology of Vallerand [46]. The Arabic version of the ICSCS has been validated for Tunisian athletes and has good psychometric properties [45]; the English version is attached as Supplementary Table S1. This tool makes it possible to measure ten coping strategies: mental imagery, thought control, expenditure of effort, search for support, relaxation, logical analysis, evacuation of unpleasant emotions, disengagement, social withdrawal, and mental distraction via 39 items rated on a 5-point Likert scale. Each strategy has four items [response scale ranging from 1 (never) to 5 (always)], except for the effort expenditure strategy, which has three items [response scale ranging from 1 (never) to 5 (always)]. The footballers were asked to answer all the items of the Arabic version of ICSCS one hour after their competition. The time to complete the ICSCS was about 15 min.

Measurements of body height were measured using a stadiometer, and weight was measured using a calibrated electronic scale (Tanita, Tokyo, Japan). BMI was calculated as body weight in kilograms divided by square height in meters. Weight status was defined according to age-specific BMI thresholds [47]. All measurements were taken by the same research group (4 investigators) directly in the stadium where the competition was taking place.

### 2.4. Statistical Analysis

The data obtained from the responses to the various items of the Arabic version of ICSCS were summarized in the form of means and standard deviation (SD) to illustrate the level of coping among footballers. Then, the scores obtained were analyzed by MANOVA on repeated measures [period (one week before Ramadan, during the last week of Ramadan, and one week after Ramadan 2021) × coping]. The Wilks Lambda test was centralized to compare the means and test the significant effect of Ramadan fasting on the coping profile in professional Tunisian footballers. All the observed differences were statistically significant when the *p*-value < 0.05 and effect sizes were calculated using eta square (η^2^ = sum of squares in group effects/total sum of squares in the ANOVA). More specifically, a total sum of squares in the ANOVA included group effect, time effect, interaction effect, and error effect in the present study. The magnitude of eta squared is explained according to Cohen’s suggestion: eta squared 0.01 as small effect; 0.06 as moderate effect; and 0.14 as large effect [48]. Data were analyzed using IBM SPSS statistics software (IBM SPSS software, France, version 21.0).

## 3. Results

The results of the MANOVA variance test demonstrated the effect of Ramadan fasting on the following coping strategies: effort expenditure [F _(11.17)_ = 3.11; *p*-value = 0.01; η2 = 0.129], seeking support [F _(11.17)_ = 1.99; *p*-value = 0.032; η^2^ = 0.112], and venting of unpleasant emotions [F _(11.17)_ = 2.31; *p*-value = 0.012; η^2^ = 0.123] (Table 2).

One week before the Ramadan fast, Tunisian male professional soccer players were using task-oriented coping strategies [Effort expenditure: 4.19 ± 0.88]; [Thought control: 3.94 ± 0.82]; [Mental Imagery: 3.83 ± 0.81]; [Logic analysis: 3.64 ± 0.89]; [Rebound: 3.34 ± 0.82]; [Support seeking: 3.31 ± 0.89]. During the last week of Ramadan, Tunisian male professional footballers adopted the following coping strategies [Social withdrawal: 4.31 ± 0.68]; [Logic analysis: 4.10 ± 0.78]; [Rebound: 3.82 ± 0.81]; [Mental Imagery: 3.72 ± 0.90]; [Disengagement: 3.71 ± 0.68]. One week after Ramadan fasting, Tunisian male professional soccer players used the following coping strategies [Logical analysis: 3.90 ± 0.81]; [Thought control: 3.88 ± 0.88]; [Disengagement: 3.82 ± 0.76]; [Mental imagery: 3.74 ± 0.84] (Table 3).

A week before Ramadan, active coping dominates the coping profile in the majority of Tunisian professional clubs. During the last week of Ramadan, footballers from most Tunisian professional clubs use the strategy of social withdrawal. One week after Ramadan, effort expenditure and social withdrawal strategies dominate the adaptation profile among Tunisian professional clubs (Table 4). The results in Table 4 present the profile of coping among the different clubs of the Tunisian professional football league before, during, and after the month of Ramadan. This mass of information gives us an in-depth idea of the mental preparation system adopted by Tunisian clubs.

## 4. Discussion

This present study showed that Tunisian professional soccer players simultaneously use positive and negative coping strategies during the fasting month of Ramadan, whereas they use active coping strategies before and after Ramadan fasting. Furthermore, the results of the current study demonstrated that there is a significant effect of Ramadan fasting on the coping strategies of the footballers. Therefore, the study findings supported the proposed hypothesis. Understanding the associations between coping strategies and Ramadan fasting can help Muslim athletes obtain possible strategies for coping with Ramadan and further help them improve sports performance. Specifically, there are conflicting findings on the effects of Ramadan on performance in general and football [25]. Some studies provided evidence regarding how Ramadan fasting associated with several aspects of performance, as well as the cognitive element of adaptation, which agree with prior research’s findings [15,16,17,23,24,25,34,49]. On the other hand, several other studies did not identify any effect of Ramadan fasting on the performance of Muslim athletes, especially for those who have developed coping strategies to cope with Ramadan fasting [7,44,50,51].

The present study revealed that task-oriented active coping was the most used to distinguish the coping pattern in most Tunisian professional soccer players before and after Ramadan fasting. This type of coping strategy, in which the athlete directly confronts the threatening situation, is positively associated with performance [42,43,44]. Although it comes from different contexts, the strategies of effort expenditure, thought control, mental imagery, logical analysis, relaxation, and seeking support remain the most used task-oriented coping strategies during Ramadan. The study of Farooq et al. [30] reported that elite footballers held negative beliefs and attitudes toward Ramadan fasting. Psychological stress in soccer players is a critical variable due to altered circadian clock and mood disturbances during Ramadan fasting [5,6,7,16]. On the other hand, the study of Zerguini et al. [32] concluded that biochemical, nutritional, subjective well-being, and performance variables were not negatively affected in young male national-level players who followed fasting of Ramadan in a controlled environment.

Although there is no special mental preparation aimed at helping Tunisian professional footballers during Ramadan, the self-adaptation that the Muslim athlete tries to use, in addition to the spiritual environment that surrounds him, all these factors help him to face the pressure imposed by the fasting system in the month of Ramadan. Studies show that social support around the athlete, along with the strength of spiritual beliefs [24], and choosing the best coping strategy may be a moderate variable in dealing with stressors that have arisen during Ramadan fasting [33]. According to Afacan [29], the adaptation strategies of Turkish professional footballers have been categorized into four dimensions: training, nutrition, lifestyle, and mental changes. It has also been determined that professional soccer players frequently use training and nutrition strategies. Meanwhile, other studies have recognized that mental preparation before fasting during Ramadan is necessary to acquire proactive coping skills [5,29,41,52]. The study of Fenneni et al. [53] confirms that experienced athletes, i.e., more trained athletes, have better-coping strategies than beginners. However, the coping methods of Ramadan fasting could be impacted by spiritual bias. Specifically, prior evidence shows that people engaging in Ramadan fasting may consider that physical discomfort was because of God’s instruction [54]. In this regard, they may not be aware of using inappropriate coping methods and result in poor physical outcomes.

### Limitations and Recomandations

In the present study, we examined the coping strategies used by footballers belonging to the Tunisian first professional league. Regarding the sample size of the current study, we focused on the main players who regularly participate in official matches. The experimental protocol adopted in the present study was carried out in three periods, before, during, and after the month of Ramadan. Therefore, submitting participants to four measures (familiarization, measures 1, 2, and 3), is considered among the limitations of this study. It was very difficult to pass the questionnaire, especially after a negative performance which can influence the credibility of the answers. In addition, measuring their height and weight for body mass index (BMI) calculation in three sessions within a short period is also one of the limitations of the current study.

This study attempts to shed light on the footballer’s relationship with Ramadan fasting in Tunisia. But the Ramadan fast still requires studies and clarifications, particularly at the level of other categories of athletes (women and young athletes, etc.) as well as at the level of other sports disciplines (handball, athletics, combat sports...). Studies in this area can also be expanded to include sedentary people. Thus, it is necessary to carry out several other studies to determine whether fasting during the month of Ramadan has a positive or negative effect on sports performance and the extent of its impact on the diet and lifestyle of the athlete in particular and sedentary people in general.

Although revealing exciting results, this study has some limitations that should be mentioned. First, the most important is that all the study measurements were taken during the Coronavirus (Covid-19) period in 2021. Second, the use of the questionnaire via the self-report method is a limitation. According to Fortes [35], the use of Likert scale-based questionnaires in repeated measures surveys can make an educational impact. A measure of the effect of dietary change during Ramadan was not available. In addition, psychobiological signals such as cortisol and testosterone were not evaluated during testing as we were unable to collect blood and urine samples during this study. It was also more appropriate for us to measure the effect of Ramadan fasting on the coping strategies of Tunisian professional football players. Lastly, we did not use any external validity control to select our participants. Therefore, the present study might have some selection bias, and the present findings might have restricted external validity.

## 5. Conclusions

This study is one of the few attempts to examine the effects of Ramadan fasting on the coping profile of fasting Tunisian professional footballers. The results showed that the adaptation profile of footballers during Ramadan is unstable and irregular, which is significantly affected by fasting, which supported the current study hypothesis. In the period of Ramadan fasting, professional soccer players use both active task-oriented and passive coping strategies toward disengagement and distraction. In contrast, they use active coping strategies before and after Ramadan fasting. Thus, from a practical point of view, integrating a mental preparation program to develop coping strategies before the Ramadan fast seems to be an appropriate intervention strategy. However, further research is warranted due to the shortcomings mentioned above.

In summary, for Muslim athletes, fasting in the month of Ramadan is generally considered an essential religious and spiritual duty. Despite the difficulties caused by fasting in the month of Ramadan on the physical and mental levels, the Muslim athlete tries to resist these difficulties by using particular coping strategies which help him to face this stressful situation.

## Figures and Tables

**Table 1:** Mean and standard deviation of descriptive research variables (n = 15, N = 180).

Club		Club 1	Club 2	Club 3	Club 4	Club 5	Club 6	Club 7	Club 8	Club 9	Club 10	Club 11	Club 12
Age	Mean ± SD	24.0 ± 4.20	23.87 ± 3.9	24.60 ± 4.4	25.33 ± 4.5	24.80 ± 4.1	25.40 ± 4.0	25.67 ± 4.3	24.93 ± 4.3	25.87 ± 3.9	25.67 ± 4.3	26.87 ± 3.8	29.47 ± 5.5
Height (cm)	Mean ± SD	180.4 ± 7.1	182.2 ± 8.3	181.2 ± 7.1	182.13 ± 8.1	180.26 ± 7.9	179.6 ± 7.1	181 ± 7.6	178.46 ± 8.1	179.53 ± 6.7	181.47 ± 7.1	179.93 ± 7.6	180.86 ± 7.2
Weight (kg)	Mean ± SD	77.8 ± 5.9	78.67 ± 6.1	77.27 ± 6.5	78.13 ± 6.4	76.2 ± 6.2	75.73 ± 6.2	77.73 ± 6.1	76.06 ± 6.7	75.2 ± 4.5	78.2 ± 5.9	77 ± 6.1	78.33 ± 6.0
BMI	Mean ± SD	23.8 ± 0.5	23.83 ± 0.5	23.51 ± 0.6	23.53 ± 0.5	23.64 ± 0.8	23.43 ± 0.6	23.70 ± 0.5	23.84 ± 0.4	23.33 ± 0.6	23.72 ± 0.4	23.73 ± 0.8	23.92 ± 0.3

SD: standard deviation; BMI: weight divided by height squared, expressed in kg/m2; Club 1: CAB; Club 2: UST; Club 3: CA; Club 4: ESS; Club 5: ASS; Club 6: CSS; Club 7: ESM; Club 8: USB; Club 9: USMO; Club 10: ASR; Club 11: EST; Club 12: OB.

**Table 2:** Inter-subject effects tests (N = 180).

**Source**	**Dependent Variable**	Before Ramadan	**Mean** **± SD**	**F**	***p*-Value**	**η^2^**	During Ramadan	**Mean** **± SD**	**F**	***p*-Value**	**η^2^**	After Ramadan	**Mean** **± SD**	**F**	***p*-Value**	**η^2^**
Footballers	MI		3.83 ± 0.81	3.54	0.001	0.170		3.72 ± 0.9	1.77	0.063	0.103		3.74 ± 0.84	3.77	0.001	0.161
	TC		3.94 ± 0.82	4.75	0.001	0.185		3.51 ± 094	1.28	0.240	0.063		3.88 ± 0.88	4.06	0.001	0.211
	EE		4.19 ± 0.88	2.34	0.010	0.139		2.38 ± 1.04	3.11	0.001	0.129		3.33 ± 1.36	30.38	0.001	0.635
	SS		3.31 ± 0.89	2.08	0.024	0.119		3.69 ± 0.89	1.99	0.032	0.112		3.52 ± 0.89	2.46	0.007	0.166
	Rx		3.34 ± 0.82	1.79	0.061	0.094		3.82 ± 0.81	1.54	0.123	0.065		3.57 ± 0.87	3.46	0.001	0.176
	LA		3.64 ± 0.89	4.97	0.001	0.235		4.10 ± 0.78	1.22	0.279	0.077		3.90 ± 0.81	2.85	0.002	0.124
	VUE		3.15 ± 1.04	18.31	0.001	0.564		2.85 ± 0.97	2.31	0.012	0.123		3.23 ± 1.03	11.59	0.001	0.454
	Di		3.15 ± 1.15	26.52	0.001	0.617		3.71 ± 0.68	0.53	0.879	0.023		3.82 ± 0.76	5.21	0.001	0.279
	SW		2.84 ± 1.02	2.64	0.004	0.143		4.31 ± 0.78	0.93	0.515	0.047		3.48 ± 1.2	20.45	0.001	0.604
	MD		2.39 ± 1.04	5.79	0.001	0.282		3.53 ± 0.82	1.30	0.229	0.037		2.85 ± 1.12	10.92	0.001	0.408

MI: Mental imagery; TC: Thought control; EE: Effort expenditure; SS: Seeking support; Rx: Relaxation; LA: Logical analysis; VUE: Venting of unpleasant emotions; Di: Disengagement; SW: Social withdrawal; MD: Mental distraction. SD: standard deviation. F: Fisher test, η2; effect sizes.

**Table 3:** Level of coping among professional footballers before, during, and after Ramadan (N = 180).

Coping Strategies	Mental Imagery	Thought Control	Effort Expenditure	Seeking Support	Relaxation	Logical Analysis	Venting of Unpleasant Emotions	Disengagement	Social Withdrawal	Mental Distraction	A	B	C
LCBR											0.001	0.001	0.001
Mean ± SD	3.83 ± 0.81	3.94 ± 0.82	4.19 ± 0.88	3.31 ± 0.89	3.34 ± 0.82	3.64 ± 0.89	3.15 ± 1.04	3.15 ± 1.15	2.84 ± 1.02	2.39 ± 1.04			
LCDR													
Mean ± SD	3.72 ± 0.9	3.51 ± 0.94	2.38 ± 1.04	3.69 ± 0.89	3.82 ± 0.81	4.10 ± 0.78	2.85 ± 0.97	3.71 ± 0.68	4.31 ± 0.78	3.53 ± 0.82			
LCAR													
Mean ± SD	3.74 ± 0.84	3.88 ± 0.88	3.33 ± 1.36	3.52 ± 0.89	3.57 ± 0.87	3.90 ± 0.81	3.23 ± 1.03	3.82 ± 0.76	3.48 ± 1.20	2.85 ± 1.12			

SD: standard deviation; LCBR: level of coping before Ramadan; LCDR: level of coping during Ramadan; LCAR: level of coping after Ramadan. A: significant differences between LCBR and LCDR; B: significant differences between LCBR and LCAR. C: significant differences between LCDR and LCAR.

**Table 4:** Level of coping among Tunisian professional clubs before, during, and after Ramadan (n = 15; N = 180).

Club		Mental Imagery	Thought Control	Effort Expenditure	Seeking Support	Relaxation	Logical Analysis	Venting of Unpleasant Emotions	Disengagement	Social Withdrawal	Mental Distraction
Level of coping before Ramadan among professional Tunisian clubs											
Club 1	Mean ± SD	3.23 ± 0.6	3.15 ± 0.7	3.62 ± 1.1	3.08 ± 0.7	3.28 ± 0.8	2.60 ± 0.6	2.41 ± 0.5	2.53 ± 0.5	3.40 ± 0.9	3.85 ± 1.0
Club 2	Mean ± SD	4.01 ± 1.1	3.48 ± 0.9	4.35 ± 0.4	3.36 ± 0.8	3.53 ± 0.7	3.91 ± 0.6	2.05 ± 0.7	2.51 ± 0.6	3.31 ± 1.4	2.51 ± 1.0
Club 3	Mean ± SD	3.96 ± 0.7	3.78 ± 0.6	3.86 ± 1.1	3.38 ± 0.9	3.55 ± 0.7	4.20 ± 0.8	2.32 ± 0.7	2.15 ± 0.7	3.23 ± 1.0	2.21 ± 1.1
Club 4	Mean ± SD	3.63 ± 0.7	4.08 ± 0.6	3.75 ± 1.1	3.30 ± 1.0	3.26 ± 0.6	3.16 ± 1.0	2.53 ± 0.9	2.63 ± 0.7	3.31 ± 0.7	2.75 ± 0.8
Club 5	Mean ± SD	3.86 ± 0.9	3.81 ± 0.8	4.15 ± 1.0	3.23 ± 0.9	3.60 ± 0.6	3.75 ± 0.9	2.80 ± 0.8	2.08 ± 0.8	2.98 ± 0.9	2.23 ± 0.8
Club 6	Mean ± SD	3.71 ± 0.6	4.03 ± 0.9	3.91 ± 1.1	3.25 ± 0.8	3.06 ± 1.0	3.66 ± 1.1	2.41 ± 0.7	2.23 ± 0.8	2.60 ± 1.2	2.02 ± 0.8
Club 7	Mean ± SD	3.46 ± 0.8	3.50 ± 0.9	4.29 ± 0.8	2.85 ± 0.9	2.85 ± 0.6	3.15 ± 0.8	3.35 ± 0.9	2.71 ± 1.1	2.66 ± 1.3	2.72 ± 1.0
Club 8	Mean ± SD	3.55 ± 0.8	3.91 ± 0.5	4.22 ± 0.8	2.85 ± 0.8	3.23 ± 0.7	3.60 ± 0.7	4.01 ± 0.5	3.80 ± 0.8	2.68 ± 1.0	2.53 ± 1.1
Club 9	Mean ± SD	3.68 ± 0.7	4.43 ± 0.7	4.49 ± 0.4	3.21 ± 0.8	3.60 ± 0.7	3.90 ± 0.6	4.08 ± 0.6	4.05 ± 0.6	2.33 ± 0.5	2.15 ± 0.7
Club 10	Mean ± SD	4.10 ± 0.6	4.40 ± 0.7	4.62 ± 0.5	3.71 ± 0.9	3.60 ± 0.9	3.85 ± 1.0	3.78 ± 0.8	4.30 ± 0.5	2.81 ± 0.8	1.65 ± 0.6
Club 11	Mean ± SD	4.62 ± 0.4	4.21 ± 0.7	4.44 ± 0.5	3.91 ± 0.9	3.55 ± 0.8	4.11 ± 0.4	4.11 ± 0.5	4.56 ± 0.6	2.50 ± 0.5	1.83 ± 0.9
Club 12	Mean ± SD	4.13 ± 0.8	4.45 ± 0.5	4.58 ± 0.5	3.61 ± 0.7	2.93 ± 1.1	3.81 ± 0.6	3.88 ± 0.7	4.23 ± 0.5	2.21 ± 0.7	2.16 ± 0.9
Level of coping during Ramadan among professional Tunisian clubs											
Club 1	Mean ± SD	3.05 ± 0.9	3.32 ± 0.9	1.84 ± 0.5	3.06 ± 0.9	3.45 ± 0.7	3.81 ± 0.6	2.88 ± 0.9	3.56 ± 0.7	4.25 ± 0.8	3.71 ± 0.8
Club 2	Mean ± SD	3.82 ± 0.6	3.55 ± 0.7	2.02 ± 1.1	3.48 ± 1.0	3.65 ± 0.6	4.13 ± 0.9	2.43 ± 0.5	3.51 ± 0.5	4.42 ± 0.7	3.48 ± 0.4
Club 3	Mean ± SD	3.75 ± 0.7	3.56 ± 0.7	2.62 ± 1.0	3.43 ± 06	3.66 ± 05	4.00 ± 1.0	2.21 ± 0.8	3.83 ± 0.4	4.42 ± 0.5	3.18 ± 0.6
Club 4	Mean ± SD	3.56 ± 0.9	3.50 ± 0.8	2.51 ± 0.8	4.00 ± 0.6	3.86 ± 0.7	3.73 ± 0.6	3.21 ± 1.0	3.75 ± 0.7	4.42 ± 0.6	3.28 ± 0.8
Club 5	Mean ± SD	4.00 ± 1.0	3.40 ± 0.8	2.24 ± 1.0	3.95 ± 0.8	4.18 ± 0.5	3.91 ± 1.0	2.43 ± 0.7	3.85 ± 0.5	4.48 ± 0.5	3.40 ± 1.2
Club 6	Mean ± SD	3.66 ± 1.1	3.85 ± 1.0	3.13 ± 1.3	3.45 ± 1.0	3.62 ± 1.0	4.08 ± 0.5	2.81 ± 0.9	3.78 ± 0.8	4.32 ± 0.7	3.98 ± 0.9
Club 7	Mean ± SD	3.96 ± 0.7	3.48 ± 1.1	2.24 ± 1.0	4.00 ± 0.9	3.91 ± 1.1	4.35 ± 0.6	2.80 ± 1.0	3.46 ± 0.9	4.22 ± 1.1	3.35 ± 1.0
Club 8	Mean ± SD	4.01 ± 1.0	3.21 ± 1.1	2.28 ± 0.9	3.75 ± 0.9	3.91 ± 0.9	4.35 ± 0.9	3.43 ± 1.0	3.71 ± 0.6	4.40 ± 0.7	3.48 ± 0.7
Club 9	Mean ± SD	4.11 ± 0.7	4.05 ± 0.8	3.26 ± 1.2	3.93 ± 0.8	3.53 ± 0.8	4.21 ± 0.7	2.65 ± 1.2	3.78 ± 0.8	3.82 ± 0.5	3.46 ± 0.8
Club 10	Mean ± SD	3.65 ± 0.9	3.70 ± 0.9	2.20 ± 1.2	4.08 ± 0.8	4.31 ± 0.8	4.45 ± 0.5	3.06 ± 0.9	3.85 ± 0.6	4.50 ± 0.9	3.70 ± 0.8
Club 11	Mean ± SD	3.75 ± 0.9	3.03 ± 1.1	2.31 ± 0.8	3.73 ± 0.9	3.88 ± 0.9	4.12 ± 0.9	3.23 ± 1.0	3.70 ± 0.6	4.08 ± 0.9	3.40 ± 0.8
Club 12	Mean ± SD	3.35 ± 1.0	3.46 ± 0.8	1.84 ± 0.5	3.41 ± 0.8	3.83 ± 0.6	3.98 ± 0.6	3.01 ± 0.8	3.70 ± 0.7	4.36 ± 0.7	3.86 ± 0.7
Level of coping after Ramadan among professional Tunisian clubs											
Club 1	Mean ± SD	3.33 ± 0.7	3.58 ± 1.0	3.93 ± 1.1	3.11 ± 0.8	2.86 ± 0.4	3.20 ± 0.9	3.40 ± 1.0	3.00 ± 1.1	2.73 ± 1.1	2.65 ± 0.9
Club 2	Mean ± SD	3.45 ± 0.9	3.88 ± 0.6	4.20 ± 0.7	2.76 ± 0.9	3.13 ± 0.9	3.61 ± 0.8	4.05 ± 0.6	3.61 ± 0.7	2.63 ± 0.9	2.88 ± 1.3
Club 3	Mean ± SD	3.45 ± 0.9	4.50 ± 0.6	4.31 ± 0.5	3.30 ± 0.8	3.41 ± 0.9	3.61 ± 0.8	3.90 ± 0.8	3.90 ± 0.6	2.75 ± 0.7	2.03 ± 0.6
Club 4	Mean ± SD	4.08 ± 0.7	4.41 ± 0.6	4.73 ± 0.2	3.63 ± 1.0	3.38 ± 0.8	3.78 ± 1.0	3.91 ± 0.8	4.38 ± 0.4	2.68 ± 1.0	1.81 ± 0.6
Club 5	Mean ± SD	4.50 ± 0.6	4.31 ± 0.6	4.60 ± 0.6	3.86 ± 0.9	3.88 ± 0.6	4.28 ± 0.3	3.95 ± 0.4	4.43 ± 0.5	2.46 ± 0.7	1.75 ± 0.7
Club 6	Mean ± SD	4.03 ± 0.7	4.38 ± 0.5	4.62 ± 0.5	3.70 ± 0.6	3.21 ± 1.1	3.88 ± 0.6	3.83 ± 0.6	4.25 ± 0.6	2.26 ± 0.8	2.13 ± 0.9
Club 7	Mean ± SD	3.03 ± 0.9	3.48 ± 0.8	2.04 ± 0.9	3.38 ± 1.1	3.75 ± 0.9	3.91 ± 0.6	3.10 ± 1.1	3.73 ± 0.7	4.16 ± 0.9	3.63 ± 0.9
Club 8	Mean ± SD	3.71 ± 0.6	3.51 ± 0.8	1.75 ± 0.8	3.70 ± 09	3.96 ± 0.7	4.18 ± 0.7	2.38 ± 0.5	3.76 ± 0.4	4.56 ± 0.6	3.70 ± 0.4
Club 9	Mean ± SD	3.78 ± 0.7	3.55 ± 0.8	2.57 ± 1.0	3.61 ± 0.7	3.81 ± 0.8	4.13 ± 1.1	2.20 ± 07	3.63 ± 0.4	4.53 ± 0.5	3.08 ± 0.6
Club 10	Mean ± SD	3.68 ± 0.9	3.66 ± 0.8	2.66 ± 0.9	3.80 ± 0.6	3.96 ± 0.5	3.68 ± 0.7	3.13 ± 1.1	3.80 ± 0.8	4.26 ± 0.8	3.38 ± 0.8
Club 11	Mean ± SD	3.83 ± 0.9	3.33 ± 1.0	2.13 ± 0.9	3.98 ± 0.7	4.10 ± 0.7	4.16 ± 1.0	2.58 ± 0.7	3.88 ± 0.5	4.48 ± 05	3.62 ± 1.2
Club 12	Mean ± SD	3.98 ± 06	3.91 ± 1.0	2.42 ± 1.0	3.36 ± 0.9	3.40 ± 0.9	4.33 ± 0.5	2.38 ± 1.0	3.48 ± 0.8	4.26 ± 0.7	3.52 ± 0.9

SD: standard deviation. Club 1: CAB; Club 2: UST; Club 3: CA; Club 4: ESS; Club 5: ASS; Club 6: CSS; Club 7: ESM; Club 8: USB; Club 9: USMO; Club 10: ASR; Club 11: EST; Club 12: OB.

## Data Availability

The dataset that supports the findings of this study is not openly available and it will be available from the corresponding author upon reasonable request.

## Supplementary Materials

The following supporting information can be downloaded at: https://www.mdpi.com/article/10.3390/healthcare11071053/s1, Table S1: Questionnaire for the assessment of coping strategies employed by athletes in competitive sport (Inventory of Competitive Sports Coping Strategies (ICSCS)).
