# Supplementary material for: The Effect of Ramadan Fasting on the Coping Strategies Used by Male Footballers Affiliated with the Tunisian First Professional League
Source: Healthcare (Basel). 2023 Apr 6;11(7):1053. doi: 10.3390/healthcare11071053 (PMC10094225; doi:10.3390/healthcare11071053)
Supplement: Supplementary file 1 [file healthcare-11-01053-s001.zip › healthcare-2260714-supplementary.pdf]

Supplementary Table S1: Questionnaire for the assessment of coping strategies employed by athletes in competitive sport.

No	Items	Never	Scarcely	Moderately	Mostly	Always
1	I visualized that I was in full control of the situation					
2	I swore (swearing, profanity, etc.) in my head or out loud to vent my anger					
3	I distanced myself from other athletes					
4	I applied myself by providing a constant effort					
5	I occupied my mind to think about something other than the competition					
6	I tried not to be intimidated by other athletes					
7	I asked for advice regarding my mental preparation					
8	I tried to relax my body					
9	I analyzed my past performance					
10	I lost all hope of being able to achieve my goal					
11	I mentally rehearsed the execution of my movements					
12	I got angry					
13	I isolated myself in a place conducive to reflection					
14	I made a strenuous effort					
15	I thought about my favorite hobbies so as not to think about the competition					
16	I tried to eliminate my doubts by thinking about positive things					
17	I asked advice from other athletes					
18	I tried to reduce my muscle tension					
19	I analyzed the weaknesses of my opponents					
20	I gave in to discouragement					
21	I imagined myself putting on a good performance					
22	I expressed my displeasure					
23	I made a void around me					
24	I gave my maximum effort					
25	I did fun things to keep my mind off the competition					
26	I replaced my negative thoughts with positive ones					
27	I confided in a trustworthy person					
28	I did relaxation exercises					
29	I thought of possible solutions to handle the situation					
30	I wanted the competition to end immediately					
31	I visualized my best performance ever					
33	I sought silence					
34	I thought about my successes rather than my mistakes					
35	I talked to someone who is able to motivate me					
36	I relaxed the muscles of my body					
37	I have analyzed the requirements of the competition					
38	I stopped believing in my ability to achieve my goal					
39	I thought of my family or my friends to distract me					
